# Effect of dietary supplementation of sea buckthorn and giloe leaf meal on the body weight gain, feed conversion ratio, biochemical attributes, and meat composition of turkey poults

**DOI:** 10.14202/vetworld.2018.93-98

**Published:** 2018-01

**Authors:** Aditya Sharma, Pankaj Kumar Shukla, Amitav Bhattacharyya, Upendra Kumar, Debashis Roy, Brijesh Yadav, Atul Prakash

**Affiliations:** 1 Department of Poultry Science, College of Veterinary Science and Animal Husbandry, Mathura - 281 001, Uttar Pradesh, India; 2 Department of Animal Nutrition, College of Veterinary Science and Animal Husbandry, Mathura - 281 001, Uttar Pradesh, India; 3 Department of Veterinary Physiology, College of Veterinary Science and Animal Husbandry, Mathura - 281 001, Uttar Pradesh, India; 4 Department of Pharmacology and Toxicology, College of Veterinary Science and Animal Husbandry, Mathura - 281 001, Uttar Pradesh, India

**Keywords:** giloe, growth, meat composition, sea buckthorn, turkey

## Abstract

**Aim:**

In the recent past, few studies have been carried out about sea buckthorn (SBT) and giloe in chicken as a part of the quest for suitable alternatives to antibiotics. However, studies in turkeys are lacking. Hence, the present study was conducted to evaluate the effects of SBT and giloe leaf meal by dietary feed supplementation in turkey poults.

**Materials and Methods:**

A total of 1-day-old turkey poults (n=84) of small white variety were distributed into four dietary treatments having three replicates each with seven birds. The study was conducted in turkey poults during 0-8 weeks of age. During the experiment, the poults were fed basal ration (28% crude protein [CP], 2800 Kcal/kg ME) T1, T2-basal ration was supplemented with SBT leaf meal powder at 0.5%, T3-basal ration was supplemented with giloe leaf meal powder at 0.5%, and T4-basal ration was fed along with supplementation of both SBT at 0.5% and giloe leaf meal powder at 0.5%.

**Results:**

T2 turkey poults had a significantly higher (p<0.01) body weight gain than T3 and T4 at 7^th^ week of age. Weekly body weight gain was significantly higher (p<0.05) in T2 than T3 during 5^th^-8^th^ week and 0-8^th^ week of the growth phase. Feed conversion ratio (FCR) was significantly better (p<0.01) in T2 than other treatment groups during 4^th^-8^th^ week phase of growth (2.09 vs. 2.36, 2.29 and 2.31). Further, FCR was significantly better (p<0.01) in T2 group as compared to other treatment groups during 0-8^th^ week of growth phase (1.95 vs. 2.21, 2.21 and 2.12). Plasma uric acid was found significantly increased (p<0.05) in T1 than T3 and T4, and alkaline phosphatase value was significantly higher (p<0.05) in T1 and T3 than T2. Zinc content of breast (pectoralis major) muscles was significantly higher (p<0.05) in T2 and T4 as compared to T1, while ether extract (EE) in thigh (ilio tibialis) muscles was significantly higher (p<0.05) in T2 as compared to the other treatment groups.

**Conclusion:**

It may be concluded that supplementation of SBT leaf meal at 0.5% may improve production performance of turkey poults. Supplementation of 0.5% SBT leaf meal may result in higher levels of zinc and EE in the breast and thigh cuts of turkey poults.

## Introduction

One of the most promising approaches for a viable alternative to antibiotics has been the exploration of the power of nature, i.e. herbs, shrubs, and trees. It involves the medicinal use of plants and their extracts as supplements for eliciting production in poultry. In fact, the properties of plants and their extract have been exploited as preservatives, flavors, digestive enhancers, and remedies from ancient time in humans and animals.

Sea buckthorn (*Hippophae rhamnoides*) (SBT) is a naturally growing shrub that is native to Eastern Europe and Asia and is found in the abundance in The Indian subcontinent, especially the North Western Himalayan regions [1]. SBT leaves contain significant amounts of proteins (11-20%), amino acids (0.73% lysine, 0.13% methionine, and cystine), minerals, folic acid, catechins, esterified sterols, triterpenols, and isoprenols [2-5]. Tannins hippophaenins A and B have been isolated from SBT leaf [6]. *Tinospora cordifolia* (giloe) is a deciduous climbing shrub found throughout tropical Asia. Leaves of the giloe are rich in protein (11.2%) and are fairly rich in calcium and phosphorus [7-10]. Few studies have been undertaken on the effect of supplementation of SBT leaf meal and giloe on the growth performance in broilers, and it was found that SBT leaf meal and giloe may be a viable proposition for improving the growth and feed conversion ratio (FCR) in broilers [2,11-15]. However, studies in turkeys are lacking.

In view of above, the present study was designed to assess the effect of dietary supplementation of SBT and giloe leaf meal alone or in combination on the body weight gain, FCR, biochemical attributes, and meat composition of turkey poults.

## Materials and Methods

### Ethical approval

Experiments were carried out in accordance with the guidelines laid down by the Institute Animal Ethics Committee for the use of poultry birds.

### Preparation of SBT and giloe leaf meal

Raw SBT leaves were procured from District Lahaul-Spiti in Himachal Pradesh, and *T. cordifolia* leaves were procured from Gahar village of District Bilaspur in Himachal Pradesh, India. Fresh leaves were ground and sun-dried in a clean and dust free environment to obtain a fine powder. The powder formed was packed in an airtight container.

### Experimental design, housing, feeding, and management

Eighty-four straight run day old turkey poults were divided into four treatment groups comprising of three replicates and seven turkey poults in each replicate. The poults were wing banded, weighed individually, and distributed randomly on uniform body weight basis in the treatment groups. The poults were housed in deep litter under continuous lighting schedule with a floor space of 1.5 ft^2^/poult till 8 weeks of age. Water was offered *ad libitum*. There were four dietary treatments: T1 - basal or control diet (turkey starter ration; NRC, 1994), T2 - basal or control diet (T1) + supplementation of 0.5% SBT leaf meal, T3 - basal or control diet (T1) + supplementation of 0.5% giloe leaf meal, and T4 - basal or control diet (T1) + supplementation of 0.5% SBT leaf meal and 0.5% giloe leaf meal. Representative samples of SBT and giloe leaf meal and turkey feed were analyzed for their nutrient composition, namely, dry matter (DM), CP, total ash, and crude fiber [16].

### Body weight gain and FCR

Weekly body weight, group feed consumption, and mortality were recorded. FCR (feed intake: Body weight gain) of 0 to 8 weeks was calculated at the end of the experiment.

### Blood biochemical attributes

Blood was collected from six turkey poults of each group at the end of the biological experiment from the wing vein with the help of heparinized and non-heparinized syringes and poured into sterile tubes. The blood samples were centrifuged for the 10-15 min at 2500 rpm. Plasma and serum were separated and stored in the refrigerator (−20°C) until analyzed. Plasma cholesterol, high-density lipoprotein (HDL) cholesterol, protein, uric acid, glutamate oxaloacetate transaminase (GOT), glutamate pyruvate transaminase (GPT), and alkaline phosphatase (ALP) were determined using commercial kits of Span Cogent Diagnostics Product, India, according to the manufacturer’s instructions. The lipid peroxidation (LPO)/malondialdehyde assay was done by the thiobarbituric acid reactive substance method [17]. Serum superoxide dismutase (SOD) activity was measured using the method as described by Madesh and Balasubramanian [18] with some modifications. In the microtiter plate method, the assay mixture in a total volume of 300 µl per well consisted of 120 µl phosphate buffer saline (PBS), 10 µl serum sample, 5 µl of 1.25 mM 3-(4,5-dimethyl-2-thiazolyl)-2,5-diphenyl-2H tetrazolium bromide (MTT), and 15 µl of freshly prepared 1 mM pyrogallol solution to be added at the end. Sample was replaced with PBS in the blank. After an incubation period of 15 min, 150 µl dimethyl sulfoxide was added and absorbance was taken in ELISA reader at 570 nm. The percent inhibition by the presence of SOD was calculated from the reduction of the MTT color formation as compared to the MTT formazan formed in the absence of SOD which was taken as 100%.

### Proximate composition of breast (pectoralis major) and thigh (ilio tibialis) muscle of turkey poults

After 8 weeks of age, 6 birds from each treatment group (3 male and 3 female) were sacrificed, and thereafter, fresh samples of breast (*pectoralis major*) and thigh (*ilio tibialis*) muscles were processed and analyzed for DM, CP, ether extract (EE), total ash, calcium, and phosphorus [16]. Ca and Zn in breast and thigh were determined by atomic absorption spectroscopy (AAS). Samples were digested [19]. 1 g of the sample was taken. 1 ml of pure nitric acid was added in 50 ml digestion tube. Sample was kept overnight at room temperature. Digestion was done slowly at low heat (<90°C) on micro digestion bench so that volume is reduced to about 0.5 ml. Double acid mixture (HNO3 and 70% HClO4, 3:1) was added to make the volume 5 ml. Repeated digestion was done, till white fumes emanated from it. Digested final volume was about 0.5 ml. Triple glass-distilled water was added to make 10 ml. The concentration of calcium and zinc was measured in AAS-400 using the formula:

Ca or Zn mg/100 g={Concentration of calcium or zinc in PPM or mg/l × volume made}/weight of sample.

### Statistical analysis

Data were subjected to one-way analysis of variance in a completely randomized design [20] using the Statistical Package for the Social Sciences [21]. Homogenous subsets were separated using Duncan’s multiple range test described by Duncan [22]. Differences among treatments were considered to be statistically significant when p≤0.05.

## Results

### Chemical composition of experimental diet

The proximate principles, i.e., DM, total ash, EE, calcium, phosphorous, protein, crude fiber, zinc content of turkey starter feed, SBL meal, and giloe leaf meal have been summarized in Table-1.

**Table-1:** Proximate analysis of turkey feed, sea buckthorn leaf, and giloe leaf.

Category	Dry matter (%)	Total ash (%)	Ether extract (%)	Calcium (%)	Phosphorous (%)	Protein (%)	Crude fiber	Zinc (%)
Turkey feed	89.29	8.93	2.57	2.03	1.33	27.7	21.2	0.0025
Sea buckthorn leaf meal	41.4	8.19	6.93	1.56	1.14	12.26	18.48	0.0028
Giloe leaf meal	52	11.2	1.79	0.28	0.58	13.87	21.54	0.0014

### Average body weight gain

Weekly body weight gain was found to increase till 8^th^ week (Table-2). T2 turkey poults had a significantly higher (p<0.01) body weight gain than T3 and T4 at 7^th^ week of age. Further, T2 poults had a numerically higher body weight gain compared to the other treatment groups throughout the experiment. In addition, weekly body weight gain was significantly better (p<0.05) in T2 than T3 during 5-8 weeks and 0-8 weeks of the growth phase (Table-3).

**Table-2:** Effect of sea buckthorn and giloe leaf meal supplementation on the average weekly body weight gain (g) of turkey poults during 0-8 weeks of age.

Treatment	1^st^ week	2^nd^ week	3^rd^ week	4^th^ week	5^th^ week	6^th^ week	7^th^ week	8^th^ week
T1	22.10	55.37	74.13	90.33	117.06	141.00	178.43^ab^	188.20
T2	32.11	61.72	75.00	99.94	114.44	154.83	196.17^b^	211.94
T3	23.44	52.29	66.33	82.14	97.41	126.67	159.16^a^	185.11
T4	31.77	54.43	72.87	95.00	113.47	124.50	161.20^a^	202.17
Pooled SEM	1.86	1.90	1.97	2.84	6.17	5.21	5.19	6.65
Sig level	NS	NS	NS	NS	NS	NS	p<0.01	NS

Means bearing different superscripts within a column differ significantly (p<0.05). NS=Not significant (p>0.05), SEM=Standard error of means

**Table-3:** Effect of sea buckthorn and giloe leaf meal supplementation on weekly body weight gain (g) of turkey poults at different phases of growth during 0-8 weeks of age.

Treatment	0-4 weeks	4-8 weeks	0-8 weeks
T1	241.93	624.70^ab^	866.63^ab^
T2	268.78	677.39^b^	946.17^b^
T3	224.21	568.34^a^	792.56^a^
T4	254.07	601.33^a^	855.40^ab^
Pooled SEM	7.67	15.18	21.30
Significant level	NS	p<0.05	p<0.05

Means bearing different superscripts within a column differ significantly (p<0.05). NS=Not significant (p>0.05), SEM=Standard error of means

### FCR

FCR was found comparatively better in T2 than other treatment groups during 0-4^th^ week phase of growth (Table-4). Thereafter, FCR was significantly better (p<0.01) in T2 than other treatment groups during 4^th^-8^th^ week phase of growth. Further, FCR was significantly better (p<0.01) in T2 group as compared to other treatment groups in 0-8^th^ week of growth phase.

**Table-4:** Effect of sea buckthorn and giloe leaf meal supplementation on feed conversion ratio of turkey poults at different phases of growth during 0-8 weeks of age.

Treatment	04 weeks	58 weeks	08 weeks
T1	2.06	2.36^b^	2.21^b^
T2	1.80	2.09^a^	1.95^a^
T3	2.14	2.29^b^	2.21^b^
T4	1.94	2.31^b^	2.12^b^
Pooled SEM	0.05	0.03	0.04
Sig level	NS	p<0.01	p<0.01

Means bearing different superscripts within a column differ significantly (p<0.05). NS=Non-significant (p>0.05), SEM=Standard error of means

### Blood biochemical parameters

Effect of SBT and giloe leaf meal feeding on total plasma protein, total plasma cholesterol, plasma uric acid, plasma GOT, GPT, and ALP was determined (Table-5). HDL, SOD (superoxide dismutase), and LPO also determined (Table-6). There was no significant difference among the treatment groups in any of the blood biochemical indices except plasma uric acid and ALP. Plasma uric acid was significantly increased (p<0.05) in control group than giloe and group having supplementation of both SBT and giloe leaf meal. Further, plasma ALP value was significantly higher (p<0.05) in control than SBT supplemented group.

**Table-5:** Effect of sea buckthorn and giloe leaf meal supplementation on blood biochemical parameters (protein, cholesterol, uric acid, alkaline phosphatase, SGOT, and SGPT) of turkey poults after 8 weeks of age.

Treatment	Protein (g/dL)	Uric acid (mg/dL)	Cholesterol (mg/dL)	AST (IU/L)	ALT (IU/L)	ALP (IU/L)
T1	3.88	7.99^b^	128.99	4.53	5.97	188.82^b^
T2	3.84	6.65^ab^	118.03	4.42	5.08	164.42^a^
T3	4.26	5.84^a^	122.01	3.98	5.53	182.38^b^
T4	4.34	6.11^a^	121.12	3.43	4.42	177.98^ab^
Pooled SEM	0.09	0.29	1.75	0.20	0.33	3.16
Sig level	NS	p<0.05	NS	NS	NS	p<0.05

Means bearing different superscripts within a column differ significantly (p<0.05). NS=Non-significant (p>0.05) SEM=Standard error of means. AST=Aspartate aminotransferase, ALT=Alanine aminotransferase, ALP=Alkaline phosphatase, SGOT=Serum glutamate oxaloacetate transaminase, SGPT=Serum glutamate pyruvate transaminase

**Table-6:** Effect of sea buckthorn and giloe leaf meal supplementation on blood biochemical parameters (HDL, SOD, and LPO) of turkey poults after 8 weeks of age.

Treatment	HDL (mg/dL)	SOD (units/mL)	LPO (nM/mL)
T1	60.61	457.87	0.19
T2	62.35	331.49	0.04
T3	64.49	376.89	0.04
T4	63.19	345.60	0.05
Pooled SEM	0.72	21.90	0.03
Sig level	NS	NS	NS

NS=Non-significant (p>0.05), SEM=Standard error of means, HDL=High-density lipoprotein, SOD=Superoxide dismutase, LPO=Lipid peroxidation

### Proximate analysis of breast (pectoralis major) muscles and thigh (ilio tibialis) muscles

**Figure-1:**
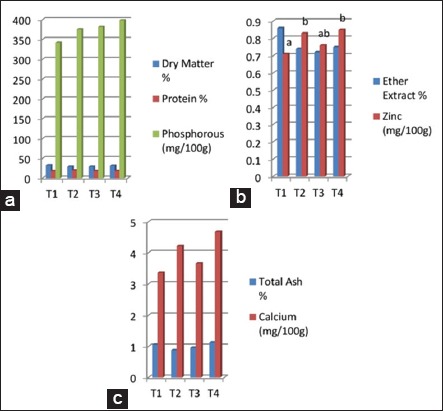
(a-c) Effect of sea buckthorn and giloe leaf meal supplementation on the proximate analysis of breast (pectoralis major) muscle of turkey poults after 8 weeks of age. Columns in the graph bearing different superscripts differ significantly (p<0.05).

**Figure-2:**
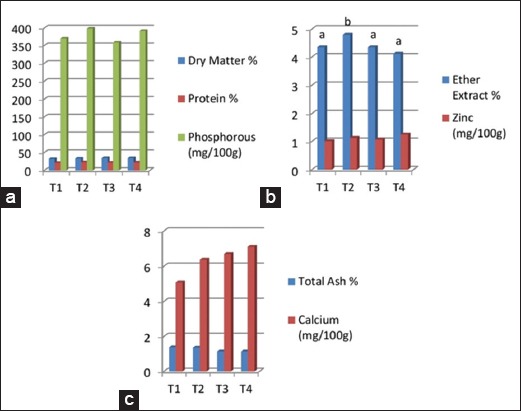
(a-c) Effect of sea buckthorn and giloe leaf meal supplementation on proximate analysis of thigh (ilio tibialis) muscle of turkey poults after 8 weeks of age. Columns in the graph bearing different superscripts differ significantly (p<0.05).

No significant difference was observed in the proximate analysis of breast (*pectoralis major*) muscles of turkey poults, except zinc percent, which was significantly higher (p<0.05) in SBT and both SBT and giloe supplemented groups as compared to control at 8 weeks of age (Figure-1a-c). Similarly, no significant difference was observed in the proximate analysis of thigh (*ilio tibialis*) muscles of turkey poults, except in percent EE, which was significantly higher (p<0.05) in SBT leaf meal supplemented group as compared to the other treatment groups (Figure-2a-c).

## Discussion

### Chemical composition of experimental diet

The proximate values of SBT leaf were in order as reported by Kashif and Ullah [5]. The proximate values of giloe leaf were in the same ranges as reported in other studies [7-10].

### Average body weight gain

It has been reported that SBT leaves contain significant amounts of proteins (11-20.7%), amino acids (0.73% lysine, 0.13% methionine, and cystine) minerals, folic acid, catechins, esterified sterols, triterpenols and isoprenols [2-5]. In our study too, the sea buckthorn leaves contained 12.26% crude protein. This might have contributed in the higher body weight gain in the SBT leaf meal supplemented group in turkey poults. Further, our results collaborate well with the findings of Wang [23], who reported that SBT positively affected the body weight of laying hens. It has also been reported that supplementation of SBT leaves extract, pulp, and oil significantly (p<0.05) increased the body weight gain [24].

### FCR

The results of the present study are in accordance with the findings of Pathak *et al*. [24] and Kaushal and Sharma [25]. It has been observed that supplementation of SBT leaves extract, pulp, and oil leads to better FCR compared to control group [24]. Further, the addition of SBT cake in poultry feed up to 30% replacement of crude protein showed better growth response and FCR [25]. In addition, FCR with diets having 5% SBT fruit residue meal was higher than control group [26].

### Blood biochemical parameters

In our study, plasma uric acid was significantly increased (p<0.05) in control group than giloe and group having supplementation of both SBT and giloe leaf meal. Further, plasma ALP value was significantly higher (p<0.05) in control than SBT supplemented group. In addition, plasma uric acid, cholesterol, aspartate aminotransferase (AST), alanine aminotransferase (ALT) and ALP, and serum LPO values were comparatively higher in the control group than other treatment groups. Ahera and Wahib [27] noted that that ethanolic extract of *T. cordifolia* increased the level of liver mitochondrial enzymes such as glutathione, catalase, and SOD but decreased the level of LPO in the liver. Therefore, it was evident that *T. cordifolia* has antioxidant activity as well as immune-modulator property through protecting the cells from the oxidative damage. Stanely *et al*. [28] reported that administration of the extract of *T. cordifolia* roots (2.5 and 50 mg/kg body weight) for 6 weeks resulted in a significant reduction of serum and tissue cholesterol, phospholipids, and free fatty acids in alloxan diabetic rats. Kavitha *et al*. [29] reported that ethanolic extract of all the parts of giloe showed significant hepatoprotective effect by a reduction in serum enzymes ALT, AST, ALP, and total bilirubin in rats.

### Proximate analysis of breast (pectoralis major) muscles and thigh (ilio tibialis) muscles

The higher zinc level in SBT and both SBT and giloe supplemented groups in breast as compared to control in our study may be due to a higher level of zinc in SBT leaves (2.83 mg/100 g). Further, the SBT leaves also had a higher EE percent (6.93%) which was reflected in the higher level of EE percent in the thigh of the SBT leaf meal supplemented group. The findings of our study fall in line with Mahmoud *et al*. [26] who reported that percentage of breast muscles and thigh muscles in live weight was not significantly different between the groups of birds with 5% SBT fruit residues. However, Li *et al*. [30] concluded that crude protein of thigh muscle was significantly increased, and EE of thigh muscle was significantly decreased by the diet supplemented with 0.1% and 0.2% flavones of SBT in broilers.

## Conclusion

The findings of the present study revealed that supplementation of SBT leaf meal at 0.5% may improve production performance of turkey poults. There was no adverse effect on the blood biochemical attributes of turkey poults subjected to SBT and giloe leaf meal supplementation at 0.5%. Supplementation of 0.5% SBT leaf meal may result in higher levels of zinc and EE in the breast and thigh cuts of turkey poults.

## Authors’ Contributions

AS conducted the experimental work and UK assisted during the experiment. AB, BY, and AP wrote the article and corrected it. PKS, AB, and DR designed the study. All authors read and approved the final manuscript.
